# NFC Smartphone-Based Electrochemical Microfluidic Device Integrated with Nanobody Recognition for C-Reactive Protein

**DOI:** 10.1021/acssensors.4c00249

**Published:** 2024-06-15

**Authors:** Suchanat Boonkaew, Katarzyna Szot-Karpińska, Joanna Niedziółka-Jönsson, Ario de Marco, Martin Jönsson-Niedziółka

**Affiliations:** † Institute of Physical Chemistry, Polish Academy of Sciences, Kasprzaka 44/52, Warsaw 01-224, Poland; ‡ Laboratory for Environmental and Life Sciences, University of Nova Gorica, Vipavska cesta 13, 5000 Nova Gorica, Slovenia

**Keywords:** nanobodies, near-field communication, smartphone, screen-printed electrode, C-reactive protein, electrochemical sensor

## Abstract

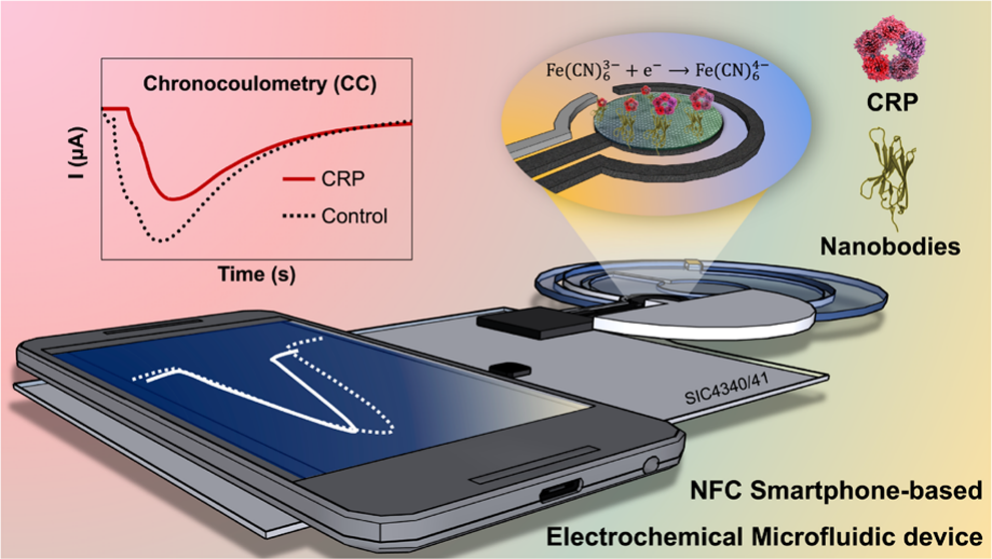

Point-of-care testing (POCT) devices play a crucial role as tools for disease diagnostics, and the integration of biorecognition elements with electronic components into these devices widens their functionalities and facilitates the development of complex quantitative assays. Unfortunately, biosensors that exploit large conventional IgG antibodies to capture relevant biomarkers are often limited in terms of sensitivity, selectivity, and storage stability, considerably restricting the use of POCT in real-world applications. Therefore, we used nanobodies as they are more suitable for fabricating electrochemical biosensors with near-field communication (NFC) technology. Moreover, a flow-through microfluidic device was implemented in this system for the detection of C-reactive protein (CRP), an inflammation biomarker, and a model analyte. The resulting sensors not only have high sensitivity and portability but also retain automated sequential flow properties through capillary transport without the need for an external pump. We also compared the accuracy of CRP quantitative analyses between commercial PalmSens4 and NFC-based potentiostats. Furthermore, the sensor reliability was evaluated using three biological samples (artificial serum, plasma, and whole blood without any pretreatment). This platform will streamline the development of POCT devices by combining operational simplicity, low cost, fast analysis, and portability.

Point-of-care testing (POCT) plays a crucial role in modern healthcare delivery, offering rapid and convenient diagnostic solutions at or near the patient’s location. Its primary advantage is the ability to provide real-time analysis, enabling healthcare providers to make immediate treatment decisions and improve patient outcomes.^1,2^ With the growing demand for personalized and timely healthcare, as evidenced during the COVID-19 pandemic, the role of POCT continues to expand, contributing to more efficient and effective healthcare.^3−6^

POCT integrated with capillary-driven microfluidic devices has gained widespread attention over the past decade.^7−9^ Traditional microfluidic devices usually require an external pump to drive fluid flow throughout the system.^10,11^ In contrast, capillary forces can be induced by the surface tension of the solution to drive the flow and devices exploiting capillary forces can operate in the absence of an external pump.^12,13^ As a result, POCT systems based on capillary-driven microfluidics have been implemented in various applications, such as the detection of heavy metals, pesticides, bacteria, viruses, biomarkers, and biomolecules.^14−18^ The most popular examples include pregnancy and COVID-19 test kits.^19,20^ These devices provide rapid results (typically within 15 min), require only a single drop of the running buffer for one-step analysis, and are inexpensive, user-friendly, and portable, but the flow control throughout the device must be accurate.^21,22^ With the aim of simplifying device operability and improving its performance, we proposed a solution based on the lamination of multiple layers of transparent PET film and double-sided adhesive (DSA) tape.^23^ This sensor facilitates automated fluid flow for washing the excess of targeted analytes and their detection by means of binders specific for C-reactive protein (CRP), but it can be adapted to accommodate other capture elements specific for CRP or, potentially, any other (soluble) biomarkers.

CRP is a biomarker that has been used for a long time to monitor systemic inflammation, infection, and more recently several other human pathologies.^24,25^ Normal CRP levels typically fall within the range of 1–3 μg mL^–1^, while high CRP levels (20–400 μg mL^–1^) are associated with inflammation, infectious diseases, cardiovascular disease (CVDs), malignant tumors, autoimmune disease, and depression.^25−28^ Although anti-CRP IgG antibodies have been traditionally used for CRP detection, their high production costs, heterogeneity after functionalization, and reliance on human or animal sources in the production process represent critical challenges.^29,30^ Consequently, alternative capture elements, including antibody fragments, peptides, aptamers, polymers, and bacteriophages, have been proposed.^23,31−34^ In the present work, we employed nanobodies previously isolated by phage display technology^35^ because they are small recombinant proteins, inexpensive to produce, and simple to engineer adopting basic molecular biology techniques. The small size of nanobodies potentially allows for a higher binding density on the electrode surface, enhancing sensor sensitivity compared to anti-CRP IgG antibodies.^35^

In recent years, near-field communication (NFC) technology has become widespread in the field of electrochemical sensors, enhancing their functionality and ease of use since enables wireless communication and data transfer at close proximity, simplifying sensor setup, calibration, and data retrieval.^36,37^ This technology allows seamless data exchange between sensors and mobile devices, providing users with an effective way to collect and analyze electrochemical data in real time. Herein, we present a smartphone-controlled NFC potentiostat integrated with a flow-through electrochemical microfluidic device via wireless communication and with the data-display conversion on Android smartphones (Figure 1a). We also compared this configuration with the performance offered by a standard potentiostat (PalmSens4) and by conventional ELISA for the evaluation CRP levels in artificial serum, plasma, and whole blood.

**Figure 1:**
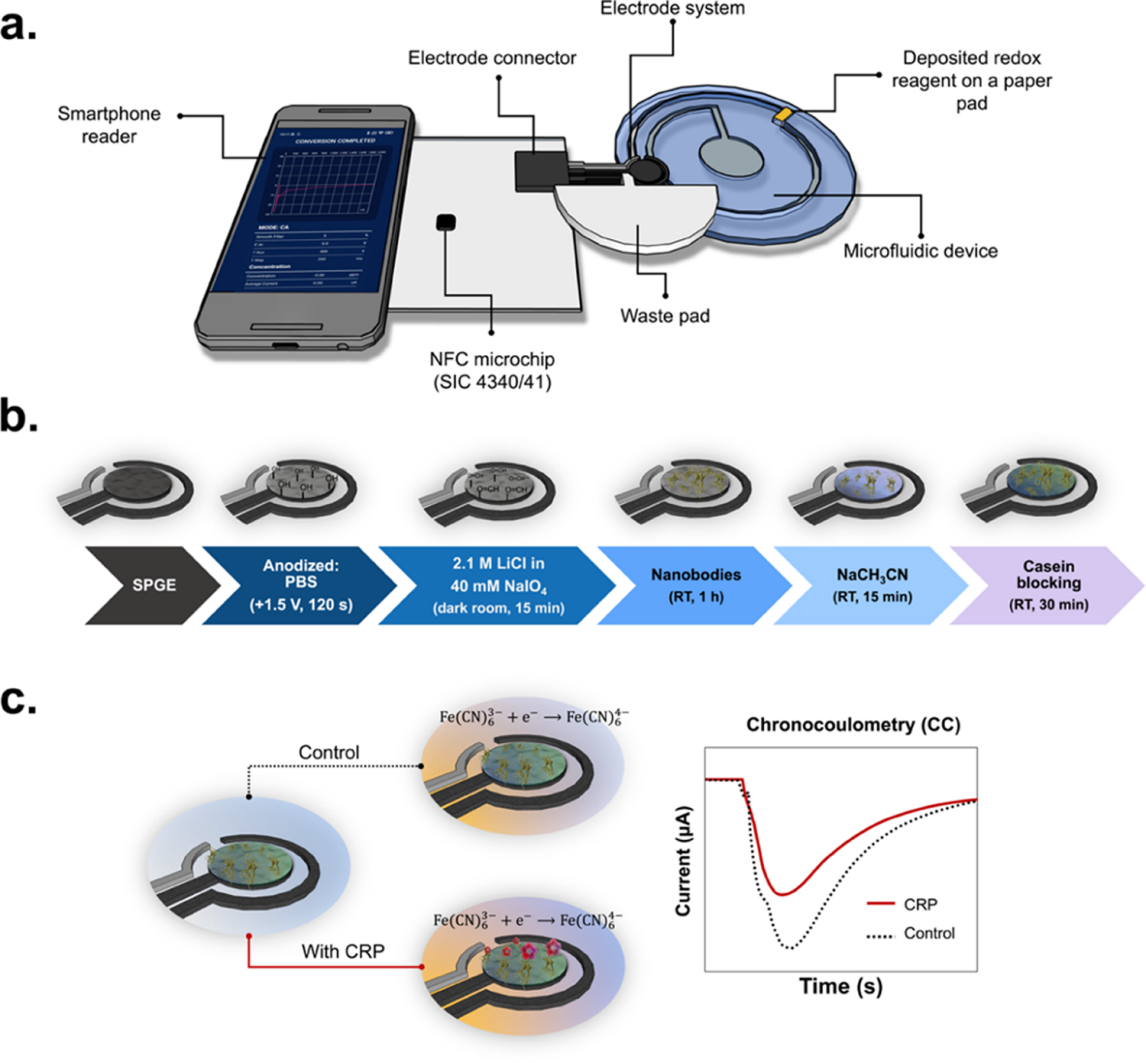
(a) Schematic illustration of the developed sensor obtained by combining the microfluidic device with a smartphone-based potentiostat. (b) Overall step-by-step modification on the screen-printed graphene electrodes (SPGEs). (c) Procedure for CRP detection using chronocoulometry (CC) measurement.

## Experimental Section

The details of materials, reagents, and equipment are presented in Supporting Information, Section 1.

### Preparation of Anti-CRP Nanobodies

The procedure relative to nanobody isolation and characterization was presented in detail in a previous report.^35^ The best clone recovered after panning (E12) was subcloned into a pET14-derived expression vector for the production of the nanobody fused to 6xHis and to SpyTag in *Escherichia coli*.^31^ Subsequently, the construct was transformed into BL21 (DE3) SOX cells for cytoplasmic expression and purified by metal affinity chromatography, as described previously.^38^ Nanobody concentration was quantified by the Bradford method, and its quality was evaluated through SDS-PAGE and gel filtration techniques.

### Electrochemical Microfluidic Device Fabrication

The details of the electrochemical microfluidic device construction, function, and testing were presented previously.^23^ Briefly, the microfluidic pattern was created by using AutoCAD software. Next, a transparent PET film (Xerox) and double-sided adhesive tape (DSA, 467MP, 3M) were laser-cut using a laser cutting method (Laser engraver GCC LaserPro, C180II) to create the flow channels, which were then integrated into a sandwich-layer configuration. The fast-flow channel had a height of 350 μm, and the delayed channel had a height of 200 μm.

The microfluidic system has an opening for loading the sample onto the electrode. After an incubation time, buffer is added to a second inlet. The buffer solution is divided into two channels: the fast-flow channel and the delayed channel. Through capillary action, the buffer flows through the fast-flow channel and reaches the electrode after ca. 4 s and washes away the sample solution together with nonbonded analyte and interfering species. The buffer flowing through the delayed channel picks up a redox molecule (K_3_Fe(CN)_6_) and arrives at the electrode after a short delay (ca. 16 s). The buffer flow continued for several minutes, during which measurements are made.

A three-electrode system consisting of a working electrode (WE, 3 mm in diameter), counter electrode (CE), and reference electrode (RE) was used to perform the electrochemical analysis. Screen-printed graphene electrodes (SPGE) were fabricated by using an in-house screen-printing method with a conductive carbon-graphene ink (Sun Chemical Company, Milan, Italy). A transparent film served as the substrate to construct SPGEs. After printing, the carbon-graphene ink was dried for 1 h at 60 °C. Then, a silver/silver chloride (Ag/AgCl, Sun Chemical Company, Milan, Italy) ink was painted on the conductive pads of the RE and dried for 1 h at 60 °C. The obtained SPGE electrode was kept under dark and dry conditions when not in use to prevent the oxidization of the Ag/AgCl. The device design and integration of the microfluidic system with a smartphone are shown in Figure 1a.

### Electrode Modification

In this study, anti-CRP nanobodies were anchored to the WE through the formation of covalent bonds. First, anodic pretreatment was performed on the SPGE, wherein a constant potential of 1.5 V versus Ag/AgCl was maintained for 120 s. This process generated hydroxyl groups (−OH) on the WE surface, as described in detail previously.^23^ Then, the electrode was rinsed with DI water and treated with a mixed solution of 2.1 M LiCl and 40 mM NaIO_4_ (5 μL) to convert the surface functional groups from hydroxyl (−OH) to aldehyde (−CHO) groups. The modified electrode was allowed to incubate in the dark for 15 min before being washed with DI water. Successively, the modified electrode was functionalized with either 1 or 10 μg mL^–1^ of anti-CRP nanobodies for 1 h at room temperature (RT) and further washed using phosphate-buffered saline (PBS, pH 7.4). The covalent binding of the nanobody on the modified electrode was obtained by means of a Schiff base reaction, resulting in the formation of an imine bond (C=N). To ensure the stability of the covalent bond, 1 mg mL^–1^ solution of NaBH_3_CN was applied to the activated electrode for 15 min, followed by PBS washing. Subsequently, 3 mg mL^–1^ of casein was added (30 min at RT) to block unsaturated residues and avoid nonspecific interactions. After a final PBS washing step, the ready-to-use SPGEs were stored in a freezer at −20 °C. An overview of the overall immobilization procedure is presented in Figure 1b.

### Electrochemical Detection of CRP

Electrochemical measurements were conducted using a PalmSens4 potentiostat/impedance analyzer (PalmSens BV, Netherlands), controlled by PStrace software version 5.9. To prevent convection effects that can influence the electrochemical current response,^39^ chronocoulometry (CC) was preferred as the method for CRP quantification. The following CC parameters were selected: *t*-equilibrium of 3 s, applied potential of 0.0 V vs Ag/AgCl, *t*-interval of 0.1 s, analysis time of 200 s, whereas the current response was measured from 16 to 180 s. For CRP detection, 4 μL of CRP solution with concentrations ranging from 0.01 ng mL^–1^ to 100 μg mL^–1^ were introduced in the sample inlet. Subsequently, following the completion of the antigen-nanobody binding reaction, 150 μL of PBS was introduced in the buffer inlet. The chronoamperometric signal was consistently recorded until the peak signal was completed. The detection principle and procedure for CRP detection using the CC measurement are shown in Figure 1c.

The NFC potentiostat used in this study was the SIC4341 (Potentiometric sensor interface chip with NFC type2) from Silicon Craft Technology PLC., Thailand. This potentiostat was integrated with a Redmi Note 10S smartphone (Xiaomi) running Android operating system. Detailed technical information and the diagram of the printed circuit board (PCB) and the actual experimental setup can be found in Table S1 and Figure S1, respectively. To control the NFC potentiostat and electrochemical parameters, perform real-time data acquisition, process data, and present electrochemical results, we used the Chemister application (NFC eco for cyclic voltammetry (CV) and chronoamperometry). The complete operative scheme relative to the combination NFC potentiostat-Android smartphone is presented in Figure S2. The parameter setting of the NFC potentiostat was identical to that used in a PalmSens4 potentiostat. Raw data were exported as a text file, and subsequent data analysis, including plotting using Microsoft Excel and the evaluation of peak height and integrated peak area, was performed using Origin Pro.

### CRP Detection in Biological Samples

Three types of samples were examined, including artificial serum (provided by Sigma-Aldrich, Warsaw, Poland), whole blood samples obtained from anonymous donors at a blood center in Warsaw, Poland, and blood plasma derived from the same whole blood samples (for details on the preparation process, see the previous report^23^). Artificial serum was diluted to 1 mg mL^–1^. The prepared artificial serum and plasma samples were subsequently spiked with varying CRP concentrations ranging from 10 ng mL^–1^ to 100 μg mL^–1^. Blood samples were used without any preparation process. The recovery efficacy of spiked CRP in artificial serum, plasma, and whole blood was calculated to assess the accuracy of the detection process.

## Results and Discussion

### Electrochemical Characterization on the NFC and Traditional Potentiostats

Electrochemical detection using a smartphone was carried out with the NFC potentiostat, a compact device with the size of a credit card, controlled by an Android system. The potentiostat integrated into the SIC4341 microchip serves essential functions, acting as a controller for the potential waveforms, as well as a real-time data collector suitable for several electrochemical applications. This system operates as a potentiostat when connected to an NFC-enabled smartphone with the Chemister application installed.

**Figure 2:**
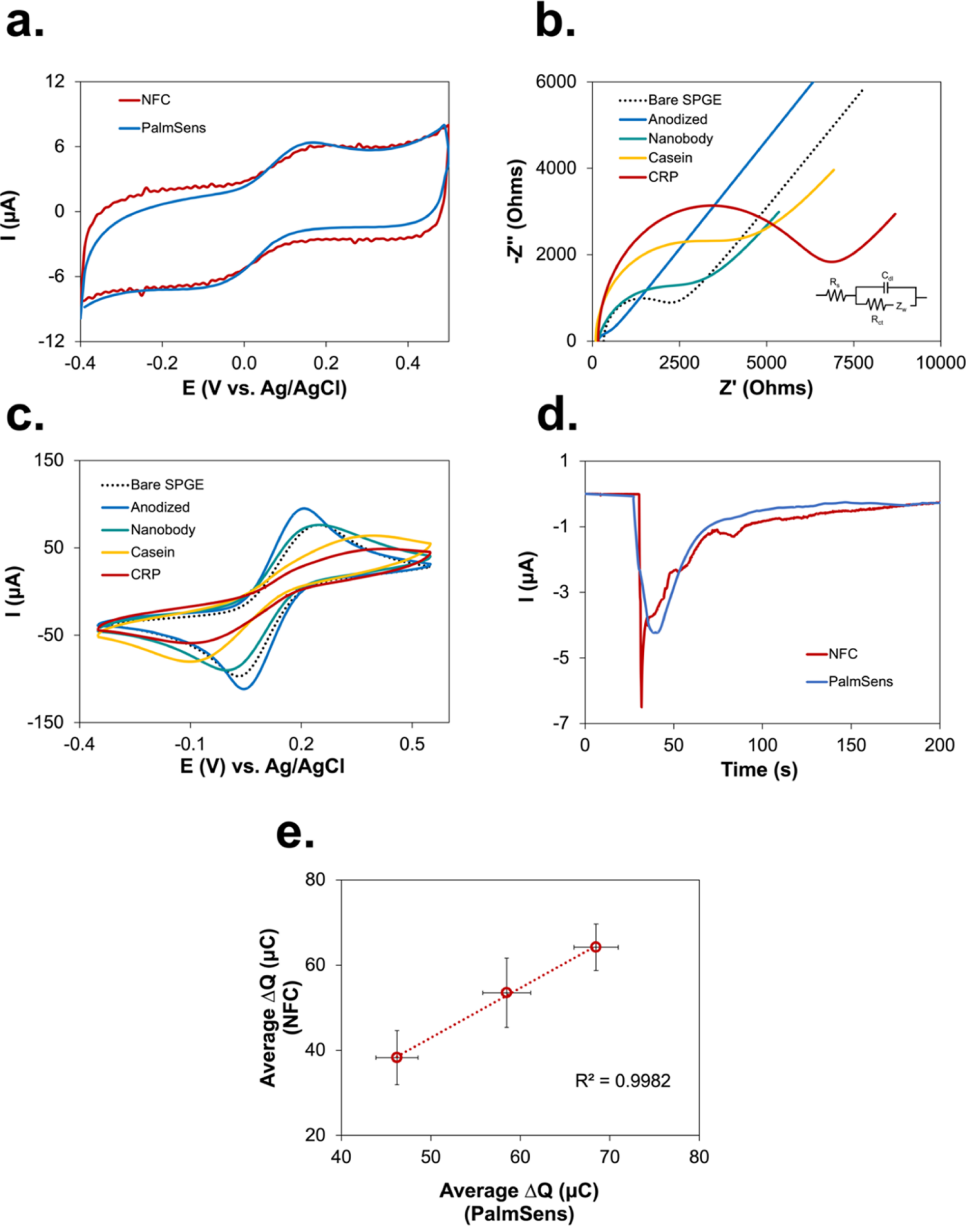
(a) CVs of 5 mM Fe(CN_6_)^3–^ and 5 mM Fe(CN_6_)^3–^ in 0.1 M KNO_3_ at a scan rate of 25 mV s^–1^ obtained from PalmSens4, used as a positive control, and the new NFC potentiostat. (b) Electrochemical impedance spectroscopy (EIS) measurement and (c) CV measurements obtained at different steps of the electrode and after incubation with CRP in a static system using 5 mM Fe(CN_6_)^3–^ and 5 mM Fe(CN_6_)^3–^ containing 0.1 M KNO_3_ at a scan rate of 100 mV s^–1^, using nanobodies as immune-capture elements. All of the Nyquist plots were fitted with the Randles circuit (inset). (d) Representation of the CC measurements obtained with PalmSens4 and NFC potentiostat using nanobody-based electrochemical biosensor in the presence of CRP. (e) Linear regression comparing the average Δ*Q* via NFC and PalmSens4 potentiostats achieved at various CRP concentrations using CC.

Initially, we examined the electrochemical performance of the NFC potentiostat in comparison with the standard lab potentiostat (PalmSens4). Cyclic voltammetry (CV) was performed using 0.5 mM [Fe(CN_6_)]^3–^ and 0.5 mM [Fe(CN_6_)]^4–^ in 0.1 M KNO_3_ to study the electroanalytical functionality of the bare SPGE on both the conventional and NFC potentiostats at the following conditions: scanned potential from −0.4 to 0.6 V vs Ag/AgCl, scan rate of 25 mV s^–1^, potential step of 10 mV, and a time step of 200 ms. Figure 2a shows the characteristic voltammograms obtained from both potentiostats and evidences their high similarity.

### Characterization of the CRP Nanobody-Modified Electrode Surface

The immobilization efficiency of anti-CRP nanobodies is a crucial step for achieving a high antigen binding specificity. To achieve this, the hydroxyl functional groups of the oxidized electrode were first modified to incorporate aldehyde groups through an oxidation reaction. Anti-CRP nanobodies were subsequently immobilized on the oxidized electrode through an imine bond (C=N). To validate the process and assess the nanobody binding capacity for the CRP target, two analytical techniques were employed: electrochemical impedance spectroscopy (EIS) and cyclic voltammetry (CV). These techniques make it possible to discriminate small variations at the interface between electrode and electrolyte, as well as to assess the electron transfer efficiency of the redox couple ([Fe(CN_6_)]^3–/4–^). The EIS Nyquist plot was fitted by using the Randles equivalent circuit, as shown in Figure 2b. The bare SPGE (dashed line) showed low electron transfer resistance (or high charge transfer resistance, denoted as high *R*_ct_), indicating lower conductivity compared with the anodized electrode (blue line). However, the introduction of the anti-CRP nanobodies (green line) onto the electrode surface induced a noticeable increase in the *R*_ct_ value. This observed increase strongly supports the successful immobilization of the immunocapture nanobody reagent E12. After the biosensor was coated with casein (yellow line), the subsequent addition of CRP (red line) triggered a significant *R*_ct_ increment. These results suggest that the immunocomplex formed between anti-CRP nanobodies and CRP affected the electron transfer of the redox solution at the electrode interface. EIS results are consistent with the CV results, as shown in Figure 2c. Specifically, the current response was progressively reduced from *I*_pa_ = 93.7 ± 3.2 μA of the anodized electrode (blue line) to *I*_pa_ = 45.8 ± 4.6 μA of the CRP signal (red line) at each successive modification step, indicating the corresponding interference of electron transfer. Both the EIS and CV results indicated the successful nanobody immobilization on the electrode surface and the nanobody’s capacity to capture CRP.

CC was also employed to quantify CRP by using the NFC potentiostat. As presented in Figure 2d, CRP quantification was obtained by measuring the peak area or the change in charge (Δ*Q*). The Δ*Q* values obtained from NFC and a conventional potentiostat using various CRP concentrations were plotted (Figure 2e) and resulted in good agreement (*R*^2^ = 0.9982) between the two potentiostats, highlighting the potential of the NFC potentiostat as a POC diagnostic tool.

Optimal analytical conditions (see SI, Section 2, and Figure S3) with the PalmsSens4 potentiostat were identified using 1 μg mL^–1^ of nanobodies (Figure S3a), 1.5 V vs Ag/AgCl of anodization potential, 120 s of anodization time, 25 mM concentration of [K_3_Fe(CN)_6_], and 40 min of incubation time (Figure S3b). Specifically, nanobody concentrations higher than 1 μg mL^–1^ introduced steric hindrance, leading to a reduced electrochemical charge response.

### CRP Detection Using Sequential Flow-Through Microfluidic Device

**Figure 3:**
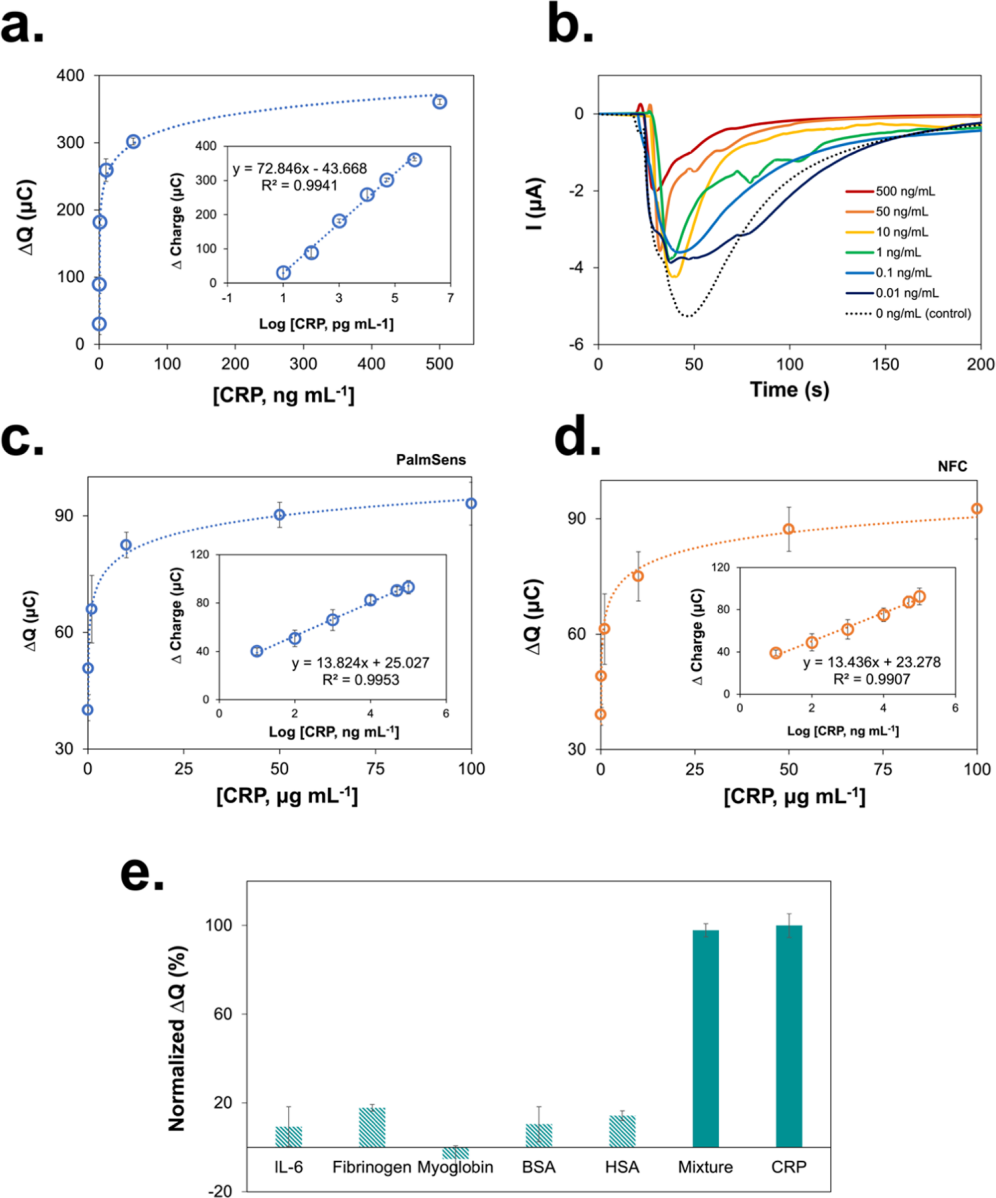
(a) Quantitative calibration plot illustrating the relationship between the change in charge (Δ*Q*) and CRP concentrations and (b) its corresponding chronoamperograms using PalmSens4 Potentiostat. (c) Calibration plot between Δ*Q* calculated using PalmSens4 and CRP concentrations performed at high anti-CRP nanobody concentrations (10 μg mL^–1^) and shorter (10 min) incubation time. (d) Same as above but using the NFC potentiostat. (e) Selectivity analysis of the diagnostic device in the presence of different proteins (interleukin-6 (IL-6), fibrinogen, myoglobin, bovine serum albumin (BSA), human serum albumin (HSA)), alone or mixed together with CRP. The error bars represent the standard deviation calculated from three replicated measurements (*n* = 3).

A comparison of analytical performances between the results obtained from PalmSens4 and the NFC potentiostat was conducted using a flow-through microfluidic device. Assay parameters were optimized to achieve the highest efficiency in terms of differentiated charge (Δ*Q* = Δ*Q*_CRP_ – Δ*Q*_control_). The analytical performance was initially examined at varying CRP concentrations with PalmSens4. In Figure 3a,3b it is evident that Δ*Q* increased as the concentration of CRP increased within the range between 0.01 and 500 ng mL^–1^. The Δ*Q* value exhibited a linear relationship with the logarithmic CRP concentration, with a correlation coefficient (*R*^2^) of 0.9941 (Figure 3a, inset) and a limit of detection (LOD) of 7.6 pg mL^–1^ (LOD = 3SD_blank_/slope), respectively. In its optimized form, the sensor detected accurately both very low CRP amounts and concentrations up to 500 ng mL^–1^. However, the upper limit is below the expected range of normal CRP levels in blood in the absence of inflammation. The long incubation time (40 min) contrasts the necessity for rapid POC diagnosis. Therefore, we tried to compensate for a shorter binding time (10 min) with an increased anti-CRP nanobody surface coverage (10 μg mL^–1^), despite the preliminary data indicating that high nanobody density could negatively affect the electrochemical current response. The data reported in Figure 3c show a linear relationship between Δ*Q* and the logarithm of CRP concentration in the range of 0.01–100 μg mL^–1^ (*R*^2^ = 0.9953), with an LOD (3SD_blank_/slope) of 1.18 ng mL^–1^ when PalmSens4 was used. Such promising results convinced us to apply the same conditions to the NFC potentiostat. As shown in Figure 3d, the linearity data were similar to those obtained with PalmSens4 and the LOD (1.79 ng mL^–1^) was just slightly higher. Summarizing, higher nanobody surface density leads to a significantly extended linear range at the cost of the sensitivity of the device to very low concentrations, therefore offering a suitable compromise between analytical accuracy in the physiologically relevant concentration range and analysis time.

Next, we evaluated the selectivity of the developed sensor for CRP using a sample in which the biomarker was mixed with equimolar amounts of common interferents, including interleukin-6 (IL-6), fibrinogen, myoglobin, bovine serum albumin (BSA), and human serum albumin (HSA). As demonstrated in Figure 3e, CRP (100 ng mL^–1^) was specifically detected in the mixed sample, whereas interferents induced negligible signals. The results indicate that the biosensor possesses a high specificity toward CRP conferred by the anti-CRP nanobodies.

The result reproducibility was assessed by comparing data collected from ten independently prepared electrochemical sensors. The standard deviation (RSD) value of 8.9% (Figure S4) is within the acceptable range, according to the Association of Official Analytical Chemists (AOAC) guidelines^40^ and confirmed the reproducibility of the proposed diagnostic approach, from the biosensor fabrication to the signal measurement.

**Figure 4:**
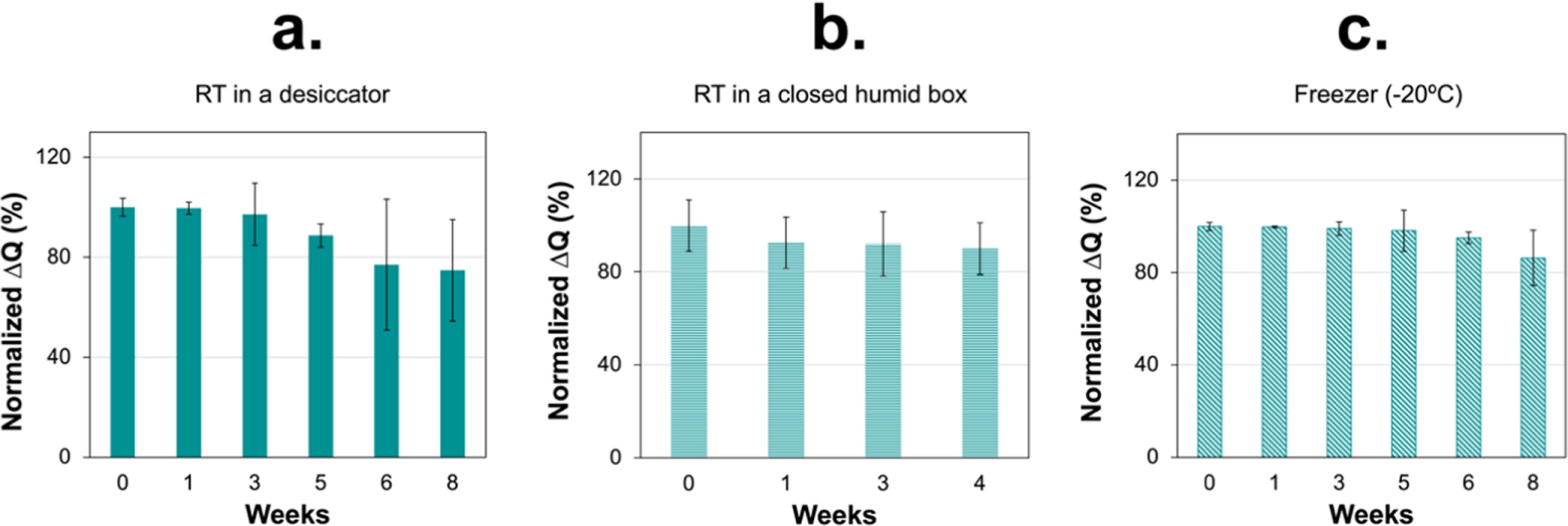
Storage stability of CRP biosensors under different conditions: (a) RT in a desiccator, (b) RT in a closed humid box, and (c) freezer (−20 °C), respectively. All measurements were calculated from three replicates (*n* = 3).

The storage stability of the biosensor was thereafter investigated by comparing three conditions selected because they are normally implemented in commercial manufacturing storage settings: (i) desiccator at room temperature (RT, 20 ± 2 °C), (ii) humid box at RT, and (iii) freezer at −20 °C. As shown in Figure 4, the biosensor maintained its performance at over 80% in the first 4 weeks under all storage conditions and, when kept in the freezer, the activity reached 86.3% even after 8 weeks (Figure 4c). These results demonstrate the superior storage stability of our device over the conventional diagnostic platforms and suggest its suitability for real-world applications characterized by challenging conditions.

Subsequently, the performance of our biosensor was compared to those of other devices designed for CRP quantification (Table S2). While its sensitivity is lower than that of some previously proposed systems, the LOD is still sufficient to detect CRP in the biologically relevant range spanning from ng mL^–1^ to μg mL^–1^. Remarkably, our device is inexpensive (less than 0.2 € per single biosensor, see Table S3 for details), rapid (complete analysis within 15 min), and portable. This makes it faster and more cost-effective than both ELISA and other electrochemical anti-CRP platforms, most of which require between 1 and 5 h.

### Clinical Samples Analysis

We finally evaluated the capacity of our biosensor to quantify CRP in clinical samples. Three conditions were considered, namely, artificial serum, plasma, and whole blood samples. Initially, artificial serum samples were spiked with CRP concentrations ranging from 10 to 500 ng mL^–1^. The calculated values for Δ*Q* and the efficiency of the proposed system were then reported as percentages of detected CRP in comparison to the theoretical concentrations. As shown in Table S4, the recovery values ranged from 91.4 to 108%, similar to the results achieved using the PalmSens4.

CRP levels in plasma samples obtained from anonymous healthy blood donors were evaluated using both the PalmSens4 potentiostat and our NFC potentiostat. The results were further compared to those obtained by ELISA (Table 1). The paired *t* test conducted on the experimental results revealed no significant difference at a 95% confidence level. Consequently, the proposed biosensor can provide accurate CRP determination in real biological samples.

**Table 1:** CRP Concentration in Plasma Samples Evaluated by Different Methods

sample	ELISA value (μg mL^–1^)^a^	detected value NFC (μg mL^–1^)	detected value PalmSens4 (μg mL^–1^)
1	1.62 ± 7.3	1.65 ± 2.8	1.73 ± 7.6
2	0.38 ± 3.3	0.40 ± 2.7	0.42 ± 1.5
3	0.73 ± 2.3	0.74 ± 0.8	0.74 ± 6.2

a It should be noted that the results were investigated using the same samples as those reported in (23); therefore, we employed the same standard ELISA values.

Finally, the proposed method was applied to the detection of CRP in whole human blood obtained from three anonymous donors. Original blood samples contained CRP amounts in the range between 0.55 and 4.20 μg mL^–1^ and were further spiked with different concentrations (from 0 to 25 μg mL^–1^) of CRP. The results of this analysis are summarized in Table 2. The recovery was found to be within the range of 82.4–120%. The errors, which were measured as percentage relative error and relative standard error (RSD), were all less than 20% for all of the tested samples. Additionally, the feasibility of this biosensor was also evaluated with an additional ten blood samples using only the PalmSens4 potentiostat, and the detailed results can be found in Table S5. Altogether, the experimental results showed that the NFC-based system integrated with the flow-through microfluidic device can correctly quantify CRP in clinically relevant biological samples without the need for pretreatment procedures and could therefore be used for the assessment of inflammation, infections caused by bacteria or viruses, and the risk of heart disease.

**Table 2:** CRP Detection in Whole Blood Samples

sample no	spiked value (μg mL^–1^)	detected value (μg mL^–1^) NFC x̅ ± SD	recovery (%)	detected value (μg mL^–1^) PalmSens4 x̅ ± SD	recovery (%)
1	0	4.20		4.06	
	0.5	4.78 ± 0.3	116	4.60 ± 1.0	108
	5	8.96 ± 1.6	95.1	9.13 ± 1.5	102
	25	26.52 ± 0.9	89.3	28.47 ± 2.7	97.6
2	0	2.05		2.35	
	0.5	2.46 ± 0.7	82.4	2.90 ± 1.1	111
	5	6.97 ± 0.9	98.4	7.82 ± 1.0	110
	25	24.98 ± 1.4	91.7	31.19 ± 0.4	115
3	0	0.55		0.77	
	0.5	1.14 ± 3.0	120	1.25 ± 1.1	95.8
	5	5.54 ± 3.3	99.9	5.36 ± 3.3	91.9
	25	25.85 ± 4.3	101	22.23 ± 1.2	85.9

## Conclusions

We have successfully developed a portable electrochemical biosensor that integrates an NFC potentiostat with a sequential flow-through microfluidic device and exploits nanobodies for the capture and quantification of CRP, providing a reliable and inexpensive diagnostic solution. Our device offers user-friendly operation, delivering the test results within 15 min at a cost of under 0.2 € per device. It has a wide linear range of detection (10 ng mL^–1^–100 μg mL^–1^) and an elevated LOD of 7.6 pg mL^–1^, and demonstrated high specificity for CRP, even in the presence of other proteins commonly found in serum samples. Its reliability was confirmed by the precise detection of CRP in artificial serum, plasma, and whole blood samples, eliminating the need for sample pretreatment steps. Importantly, this configuration can be potentially applied to any soluble biomarker by simply exchanging the recognition element used to capture the antigens. Thus, it offers an alternative and economically accessible method for the detection of any biomarker, particularly in settings where advanced clinical equipment is lacking.

## Data Availability

Data used for this article are available at the RepOD repository.^41^

## Supporting Information Available

Additional experimental details, scheme of the NFC potentiostat setup, cost breakdown of device, and comparison of literature CRP sensors (PDF)

## References

[ref1] da Silva E. T. S. G.; Souto D. E. P.; Barragan J. T. C.; de F. Giarola J.; de Moraes A. C. M.; Kubota L. T. Electrochemical Biosensors in Point-of-Care Devices: Recent Advances and Future Trends. ChemElectroChem 2017, 4 (4), 778–794. 10.1002/celc.201600758.

[ref2] Madhurantakam S.; Muthukumar S.; Prasad S. Emerging Electrochemical Biosensing Trends for Rapid Diagnosis of COVID-19 Biomarkers as Point-of-Care Platforms: A Critical Review. ACS Omega 2022, 7 (15), 12467–12473. 10.1021/acsomega.2c00638. 35474766 PMC9026073

[ref3] Johnston M.; Ates H. C.; Glatz R. T.; Mohsenin H.; Schmachtenberg R.; Göppert N.; Huzly D.; Urban G. A.; Weber W.; Dincer C. Multiplexed Biosensor for Point-of-Care COVID-19 Monitoring: CRISPR-Powered Unamplified RNA Diagnostics and Protein-Based Therapeutic Drug Management. Mater. Today 2022, 61, 129–138. 10.1016/j.mattod.2022.11.001. PMC9643339 36405570

[ref4] Broughton J. P.; Deng X.; Yu G.; Fasching C. L.; Servellita V.; Singh J.; Miao X.; Streithorst J. A.; Granados A.; Sotomayor-Gonzalez A.; Zorn K.; Gopez A.; Hsu E.; Gu W.; Miller S.; Pan C.-Y.; Guevara H.; Wadford D. A.; Chen J. S.; Chiu C. Y. CRISPR–Cas12-Based Detection of SARS-CoV-2. Nat. Biotechnol. 2020, 38 (7), 870–874. 10.1038/s41587-020-0513-4. 32300245 PMC9107629

[ref5] Yakoh A.; Pimpitak U.; Rengpipat S.; Hirankarn N.; Chailapakul O.; Chaiyo S. Paper-Based Electrochemical Biosensor for Diagnosing COVID-19: Detection of SARS-CoV-2 Antibodies and Antigen. Biosens. Bioelectron. 2021, 176, 112912 10.1016/j.bios.2020.112912. 33358057 PMC7746088

[ref6] Kumar A.; Parihar A.; Panda U.; Parihar D. S. Microfluidics-Based Point-of-Care Testing (POCT) Devices in Dealing with Waves of COVID-19 Pandemic: The Emerging Solution. ACS Appl. Bio Mater. 2022, 5 (5), 2046–2068. 10.1021/acsabm.1c01320. 35473316

[ref7] Yang S.-M.; Lv S.; Zhang W.; Cui Y. Microfluidic Point-of-Care (POC) Devices in Early Diagnosis: A Review of Opportunities and Challenges. Sensors 2022, 22 (4), 1620 10.3390/s22041620. 35214519 PMC8875995

[ref8] Xie Y.; Dai L.; Yang Y. Microfluidic Technology and Its Application in the Point-of-Care Testing Field. Biosens. Bioelectron.: X 2022, 10, 100109 10.1016/j.biosx.2022.100109. 35075447 PMC8769924

[ref9] Tang R.; Yang H.; Choi J. R.; Gong Y.; You M.; Wen T.; Li A.; Li X.; Xu B.; Zhang S.; Mei Q.; Xu F. Capillary Blood for Point-of-Care Testing. Crit. Rev. Clin. Lab. Sci. 2017, 54 (5), 294–308. 10.1080/10408363.2017.1343796. 28763247

[ref10] Binsley J. L.; Martin E. L.; Myers T. O.; Pagliara S.; Ogrin F. Y. Microfluidic Devices Powered by Integrated Elasto-Magnetic Pumps. Lab Chip 2020, 20 (22), 4285–4295. 10.1039/D0LC00935K. 33094306 PMC7654506

[ref11] Bogdanowicz R.; Jönsson-Niedziółka M.; Vereshchagina E.; Dettlaff A.; Boonkaew S.; Pierpaoli M.; Wittendorp P.; Jain S.; Tyholdt F.; Thomas J.; Wojcik P. Microfluidic Devices for Photo-and Spectroelectrochemical Applications. Curr. Opin. Electrochem. 2022, 36, 101138 10.1016/j.coelec.2022.101138.

[ref12] Xie Y.; Xu X.; Wang J.; Lin J.; Ren Y.; Wu A. Latest Advances and Perspectives of Liquid Biopsy for Cancer Diagnostics Driven by Microfluidic On-Chip Assays. Lab Chip 2023, 23 (13), 2922–2941. 10.1039/D2LC00837H. 37291937

[ref13] Mark D.; Haeberle S.; Roth G.; von Stetten F.; Zengerle R. Microfluidic Lab-on-a-Chip Platforms: Requirements, Characteristics and Applications. Chem. Soc. Rev. 2010, 39 (3), 1153–1182. 10.1039/b820557b. 20179830

[ref14] Zhou W.; Dou M.; Timilsina S. S.; Xu F.; Li X. Recent Innovations in Cost-Effective Polymer and Paper Hybrid Microfluidic Devices. Lab Chip 2021, 21 (14), 2658–2683. 10.1039/D1LC00414J. 34180494 PMC8360634

[ref15] Clark K. M.; Schenkel M. S.; Pittman T. W.; Samper I. C.; Anderson L. B. R.; Khamcharoen W.; Elmegerhi S.; Perera R.; Siangproh W.; Kennan A. J.; Geiss B. J.; Dandy D. S.; Henry C. S. Electrochemical Capillary Driven Immunoassay for Detection of SARS-CoV-2. ACS Meas. Sci. Au 2022, 2 (6), 584–594. 10.1021/acsmeasuresciau.2c00037. 36570470 PMC9469961

[ref16] Pungjunun K.; Praphairaksit N.; Chailapakul O. A Facile and Automated Microfluidic Electrochemical Platform for the In-Field Speciation Analysis of Inorganic Arsenic. Talanta 2023, 265, 124906 10.1016/j.talanta.2023.124906. 37451117

[ref17] Sierra T.; Jang I.; Noviana E.; Crevillen A. G.; Escarpa A.; Henry C. S. Pump-Free Microfluidic Device for the Electrochemical Detection of A1-Acid Glycoprotein. ACS Sens. 2021, 6 (8), 2998–3005. 10.1021/acssensors.1c00864. 34350757

[ref18] Jang I.; Kang H.; Song S.; Dandy D. S.; Geiss B. J.; Henry C. S. Flow Control in a Laminate Capillary-Driven Microfluidic Device. Analyst 2021, 146 (6), 1932–1939. 10.1039/D0AN02279A. 33492316 PMC7990706

[ref19] Samper I. C.; Sánchez-Cano A.; Khamcharoen W.; Jang I.; Siangproh W.; Baldrich E.; Geiss B. J.; Dandy D. S.; Henry C. S. Electrochemical Capillary-Flow Immunoassay for Detecting Anti-SARS-CoV-2 Nucleocapsid Protein Antibodies at the Point of Care. ACS Sens. 2021, 6 (11), 4067–4075. 10.1021/acssensors.1c01527. 34694794 PMC8565458

[ref20] Zhu X.; Wang X.; Li S.; Luo W.; Zhang X.; Wang C.; Chen Q.; Yu S.; Tai J.; Wang Y. Rapid, Ultrasensitive, and Highly Specific Diagnosis of COVID-19 by CRISPR-Based Detection. ACS Sens. 2021, 6 (3), 881–888. 10.1021/acssensors.0c01984. 33645226

[ref21] Andryukov B. G. Six Decades of Lateral Flow Immunoassay: From Determining Metabolic Markers to Diagnosing COVID-19. AIMS Microbiol. 2020, 6 (3), 280–304. 10.3934/microbiol.2020018. 33134745 PMC7595842

[ref22] Perju A.; Wongkaew N. Integrating High-Performing Electrochemical Transducers in Lateral Flow Assay. Anal. Bioanal. Chem. 2021, 413 (22), 5535–5549. 10.1007/s00216-021-03301-y. 33913001 PMC8410735

[ref23] Boonkaew S.; Szot-Karpińska K.; Niedziółka-Jönsson J.; Pałys B.; Jönsson-Niedziółka M. Point-of-Care Testing for C-Reactive Protein in a Sequential Microfluidic Device. Sens. Actuators, B 2023, 397, 134659 10.1016/j.snb.2023.134659.

[ref24] Sproston N. R.; Ashworth J. J. Role of C-Reactive Protein at Sites of Inflammation and Infection. Front. Immunol. 2018, 9, 342848 10.3389/fimmu.2018.00754. PMC5908901 29706967

[ref25] Plebani M. Why C-Reactive Protein Is One of the Most Requested Tests in Clinical Laboratories?. Clin. Chem. Lab. Med. 2023, 61 (9), 1540–1545. 10.1515/cclm-2023-0086. 36745137

[ref26] Sonuç Karaboğa M. N.; Sezgintürk M. K. A Novel Silanization Agent Based Single Used Biosensing System: Detection of C-Reactive Protein as a Potential Alzheimer’s Disease Blood Biomarker. J. Pharm. Biomed. Anal. 2018, 154, 227–235. 10.1016/j.jpba.2018.03.016. 29558723

[ref27] Kim K.-W.; Kim B.-M.; Moon H.-W.; Lee S.-H.; Kim H.-R. Role of C-Reactive Protein in Osteoclastogenesis in Rheumatoid Arthritis. Arthritis Res. Ther. 2015, 17 (1), 41 10.1186/s13075-015-0563-z. 25889630 PMC4372175

[ref28] Ali N. Elevated Level of C-Reactive Protein May Be an Early Marker to Predict Risk for Severity of COVID-19. J. Med. Virol. 2020, 92 (11), 2409–2411. 10.1002/jmv.26097. 32516845 PMC7301027

[ref29] Bryan T.; Luo X.; Bueno P. R.; Davis J. J. An Optimised Electrochemical Biosensor for the Label-Free Detection of C-Reactive Protein in Blood. Biosens. Bioelectron. 2013, 39 (1), 94–98. 10.1016/j.bios.2012.06.051. 22809521

[ref30] Chames P.; Van Regenmortel M.; Weiss E.; Baty D. Therapeutic Antibodies: Successes, Limitations and Hopes for the Future. Br. J. Pharmacol. 2009, 157 (2), 220–233. 10.1111/j.1476-5381.2009.00190.x. 19459844 PMC2697811

[ref31] Oloketuyi S.; Mazzega E.; Zavašnik J.; Pungjunun K.; Kalcher K.; Marco A.; de Mehmeti E. Electrochemical Immunosensor Functionalized with Nanobodies for the Detection of the Toxic Microalgae Alexandrium Minutum Using Glassy Carbon Electrode Modified with Gold Nanoparticles. Biosens. Bioelectron. 2020, 154, 112052 10.1016/j.bios.2020.112052. 32056958

[ref32] Szot-Karpińska K.; Kudła P.; Orzeł U.; Narajczyk M.; Jönsson-Niedziółka M.; Pałys B.; Filipek S.; Ebner A.; Niedziółka-Jönsson J. Investigation of Peptides for Molecular Recognition of C-Reactive Protein–Theoretical and Experimental Studies. Anal. Chem. 2023, 95, 14475 10.1021/acs.analchem.3c03127. 37695838 PMC10535004

[ref33] Yang H. J.; Kim M. W.; Raju C. V.; Cho C. H.; Park T. J.; Park J. P. Highly Sensitive and Label-Free Electrochemical Detection of C-Reactive Protein on a Peptide Receptor–gold Nanoparticle–black Phosphorous Nanocomposite Modified Electrode. Biosens. Bioelectron. 2023, 234, 115382 10.1016/j.bios.2023.115382. 37178497

[ref34] Szot-Karpińska K.; Kudła P.; Szarota A.; Narajczyk M.; Marken F.; Niedziółka-Jönsson J. CRP-Binding Bacteriophage as a New Element of Layer-by-Layer Assembly Carbon Nanofiber Modified Electrodes. Bioelectrochemistry 2020, 136, 107629 10.1016/j.bioelechem.2020.107629. 32818758

[ref35] Oloketuyi S.; Bernedo R.; Christmann A.; Borkowska J.; Cazzaniga G.; Schuchmann H. W.; Niedziółka-Jönsson J.; Szot-Karpińska K.; Kolmar H.; de Marco A. Native Llama Nanobody Library Panning Performed by Phage and Yeast Display Provides Binders Suitable for C-Reactive Protein Detection. Biosensors 2021, 11 (12), 496 10.3390/bios11120496. 34940253 PMC8699515

[ref36] Beck J. J.; Alenicheva V.; Rahn K. L.; Russo M. J.; Baldo T. A.; Henry C. S. Evaluating the Performance of an Inexpensive, Commercially Available, NFC-Powered and Smartphone Controlled Potentiostat for Electrochemical Sensing. Electroanalysis 2023, 35 (6), e202200552 10.1002/elan.202200552.

[ref37] Pungjunun K.; Yakoh A.; Chaiyo S.; Siangproh W.; Praphairaksit N.; Chailapakul O. Smartphone-Based Electrochemical Analysis Integrated with NFC System for the Voltammetric Detection of Heavy Metals Using a Screen-Printed Graphene Electrode. Microchim. Acta 2022, 189 (5), 191 10.1007/s00604-022-05281-x. 35420315

[ref38] Veggiani G.; de Marco A. Improved Quantitative and Qualitative Production of Single-Domain Intrabodies Mediated by the Co-Expression of Erv1p Sulfhydryl Oxidase. Protein Expression Purif. 2011, 79 (1), 111–114. 10.1016/j.pep.2011.03.005. 21421053

[ref39] Amatore C.; Pebay C.; Sella C.; Thouin L. Mass Transport at Microband Electrodes: Transient, Quasi-Steady-State, and Convective Regimes. ChemPhysChem 2012, 13 (6), 1562–1568. 10.1002/cphc.201100942. 22411777

[ref40] International A. AOAC Peer-Verified Methods Program: Manual on Policies and Procedures; Association of Official Analytical Chemists, 1993.

[ref41] Jönsson-Niedziolka M.; Boonkaew S. Electrochemical Data for NFC Smartphone-Based Electrochemical Microfluidic Device Integrated with Nanobody Recognition for C-Reactive Protein, RepOD – Repository for Open Data, 2024. https://doi.org/10.18150/M8JBP9 (accessed Feb 01, 2024). 10.1021/acssensors.4c00249 PMC11217940 38877998

