# Supplementary material for: NFC Smartphone-Based Electrochemical Microfluidic Device Integrated with Nanobody Recognition for C-Reactive Protein
Source: ACS Sens. 2024 Jun 15;9(6):3066–74. doi: 10.1021/acssensors.4c00249 (PMC11217940; doi:10.1021/acssensors.4c00249)
Supplement: Supplementary file 1 — se4c00249_si_001.pdf [file se4c00249_si_001.pdf]

Supporting information

NFC Smartphone-based electrochemical microfluidic device integrated with nanobody recognition for C-reactive protein

Suchanat Boonkaew[a*], Katarzyna Szot-Karpińska[a], Joanna Niedziółka-Jönsson[a], Ario de Marco[b], Martin Jönsson-Niedziółka[a*]

[a]*Institute of Physical Chemistry, Polish Academy of Sciences, Kasprzaka 44/52, Warsaw 01-224, Poland*

[b]*Laboratory for Environmental and Life sciences, University of Nova Gorica, Vipavska cesta 13, 5000 Nova Gorica, Slovenia*

*Corresponding author: Institute of Physical Chemistry, Polish Academy of Sciences, Kasprzaka 44/52, Warsaw 01-224, Poland, E-mail: martinj@ichf.edu.pl (MJN), suchanat.boonk@gmail.com (SB)

Table of contents

		Page
1.	Experimental section	3
2.	The printed circuit board (PCB) Specification	5
3.	Overall operation steps using an NFC potentiostat with a smartphone	6
4.	Optimization of assay conditions	7
5.	Reproducibility	8
6.	Comparison of analytical performance	9
7.	Estimated cost breakdown of the proposed sensor	11
8.	%Recovery determined by the proposed device in spiked artificial serum samples	12
9.	%Recovery determined by the proposed device in spiked whole blood samples	12

1. Experimental

1.1 Materials and reagents

All reagents used are analytical reagent grade and used as received. C-reactive protein (CRP) was purchased from Biorbyt (Cambridge, United Kingdom). Stock standard solutions of CRP and anti-CRP nanobody were prepared and diluted to the desired concentration with phosphate buffer saline (PBS, pH 7.4), then stored at -20°C until use. Whole human blood samples from healthy, anonymous donors were obtained from the blood center in Warsaw, Poland (no ethics permission required for anonymous donors).

Phosphate buffer saline (PBS) tablet (0.01 M phosphate buffer contains 0.0027 M potassium chloride and 0.137 M sodium chloride, pH 7.4 at 25°C), albumin from human serum, fibrinogen, interleukin-6 (IL-6), myoglobin, casein from bovine milk, potassium hexacyanoferrate (III) ($K_3[Fe(CN)_6]$), potassium nitrate (KNO_3), sodium cyanoborohydride ($NaCNBH_3$), lithium chloride (LiCl), and sodium periodate ($NaIO_4$) were purchased from Sigma–Aldrich (Warsaw, Poland). Graphene and silver/silver chloride (Ag/AgCl) inks were obtained from Sun Chemical company (Milan, Italy). Filter paper grade no.1 was purchased from Whatman international Ltd. (Warsaw, Poland). Hydrophilic transparent PET film (Tesa 2580) was purchased from Synctec Corporation Co.,Ltd. (Nakornpathom, Thailand). Transparency PET film was obtained from Xerox company (Warsaw, Poland). Double-sided adhesive tape (DSA, 467MP) was purchased from 3M company (Warsaw, Poland).

1.2 Apparatus

All electrochemical measurements were carried out using a PalmSens4 potentiostat (PalmSens BV, Netherlands) unless mentioned that the NFC potentiostat was used. In all cases, chronocoulometry (CC) and cyclic voltammetry (CV) were performed at room temperature (20 ± 2 °C) with the following settings for CC: potential at 0.0 V vs. Ag/AgCl, t-equilibrium at 3 s, t-interval at 0.1 s, and t-run at 200 s. For CV, the potential range was

scanned from -0.5 to +0.5 V vs. Ag/AgCl. The NFC potentiostat used in this study was the SIC4341 (Potentiometric sensor interface chip with NFC type2) from Silicon Craft Technology PLC., Thailand. Near-field communication was measured via a Redmi NS10 (Warsaw, Poland). All of the experiments were performed in at least triplicate using 5 mM $[Fe(CN)_6]^{3-}$ in 0.1 M KCl as a redox solution.

1.3 Screen-printed graphene electrode (SPGE) fabrication

The transparency film served as the substrate to construct the SPGE. The process involved applying carbon-graphene ink on a mask pattern of the electrode system and using a squeegee to create the working electrode (WE, diameter of 3 mm), the counter electrode (CE), and the conductive pad for reference electrode (RE). Next, the obtained electrodes were cured in the oven at 60°C for 1 h. Afterwards, silver/silver chloride (Ag/AgCl) was painted on the conductive pads of the RE and also cured in the oven for 1 h. The SPGE electrode was stored in dark and dry conditions when not in use to prevent the oxidization of the Ag/AgCl.

1.4 Sample preparation

Artificial serum was prepared in PBS at pH 7.4. The blood samples could be used without any preparation methods. The plasma samples were prepared following the protocol from our previous study[1]. Briefly, blood samples were collected from the blood donors, and the clot was removed by centrifugation at 2000 g for 10 min. Then, the supernatant (or designated plasma) was stored in the refrigerator at -20ºC when not in use.

Table S1 The printed circuit board (PCB) Specification

**PCB Dimension**	86 mm x 55 mm x 0.8 mm
**Sensor electrode channel**	Selectable 2/3 electrode system<br>Configurable electrode channel
**Bias potential Range**	-0.8 to +0.8 V<br>VRE, VWE range 0.4 V to 1.2 V<br>Minimum step size 5 mV
**Measurement Current Range (selectable)**	± 2.5 µA full scale<br>± 20 µA full scale
**Current Accuracy**	± 5 nA for ± 2.5 µA range<br>± 20 nA for ± 20 µA range
**Operating temperature**	0 °C to 55 °C
**Storage temperature**	-40 °C to 85 °C
**Compatible Analysis Technique**	Amperometry, voltammetry

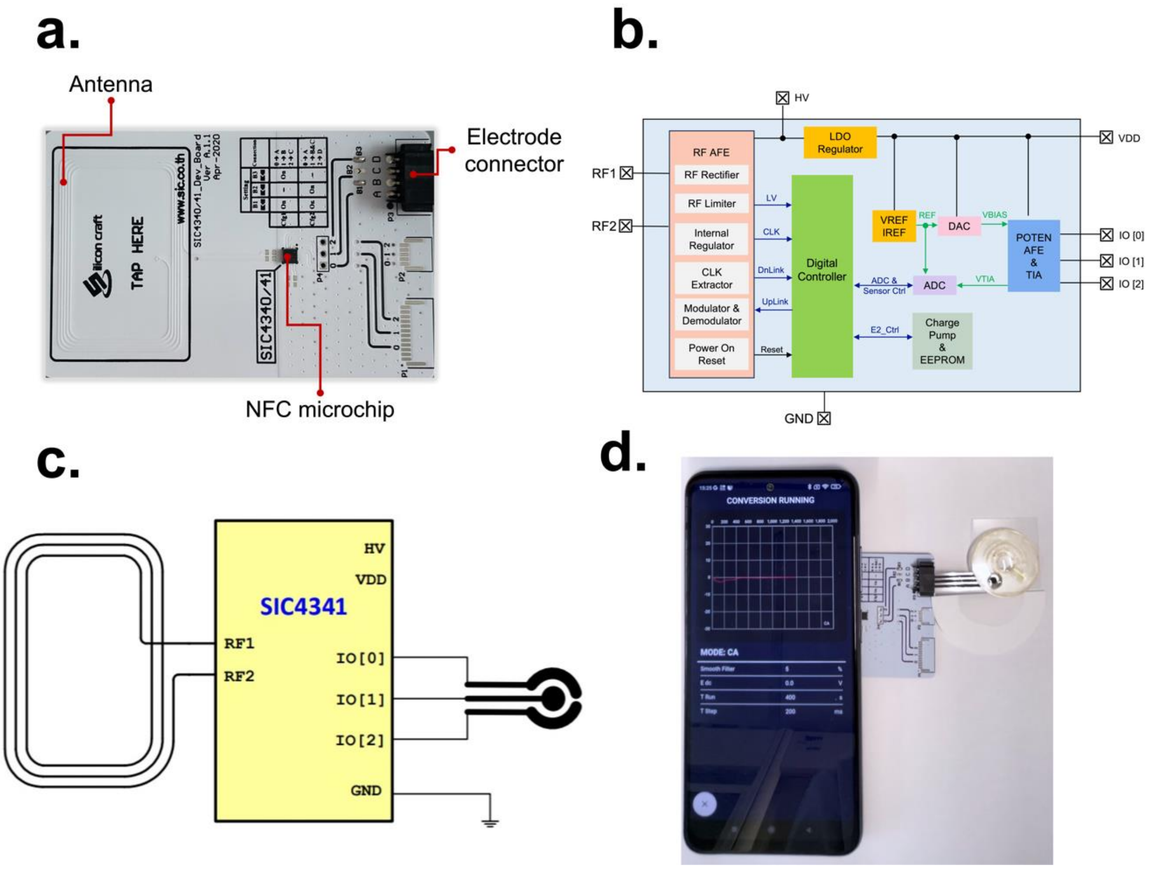

Figure S1. (a) The components of the NFC potentiostat consist of a planar antenna, the NFC microchip (SIC4340/41), and a connector port for the electrode system. (b) Functional block

diagram for the NFC potentiostat, (c) basic connections of the SIC4341with a 3-electrode system (Figures adapted from the Silicon Craft Technology PLC datasheet and previous reports [2,3]), and (d) an image showing the actual set up of the proposed system.

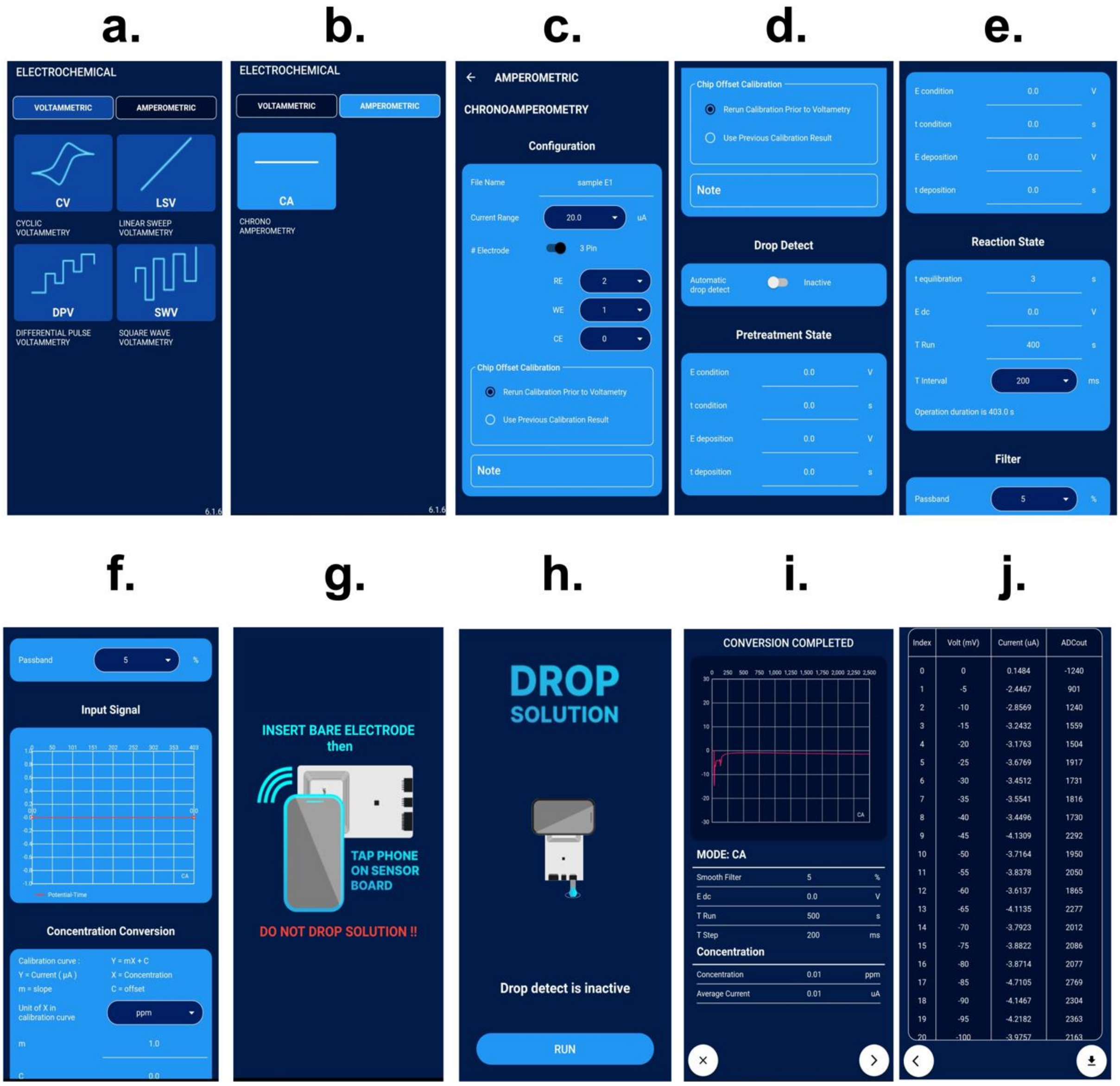

Figure S2. Images showing the overall operation steps using an NFC potentiostat with an Android smartphone. a. Shows the voltammetry options of the Chemister application for the NFC potentiostat on a smartphone running the Android operative system. b. Shows the amperometry options. c-f. show the setting screens for chronoamperometry. g. shows the screen for connecting the sensor to the potentiostat and the potentiostat to the phone. h. tells

us to apply the sample drop on the SPE. i. shows the chronoamperogram after measurements, and j. shows the associated data. The data is saved in the phone memory and can be exported using USB or wireless connections.

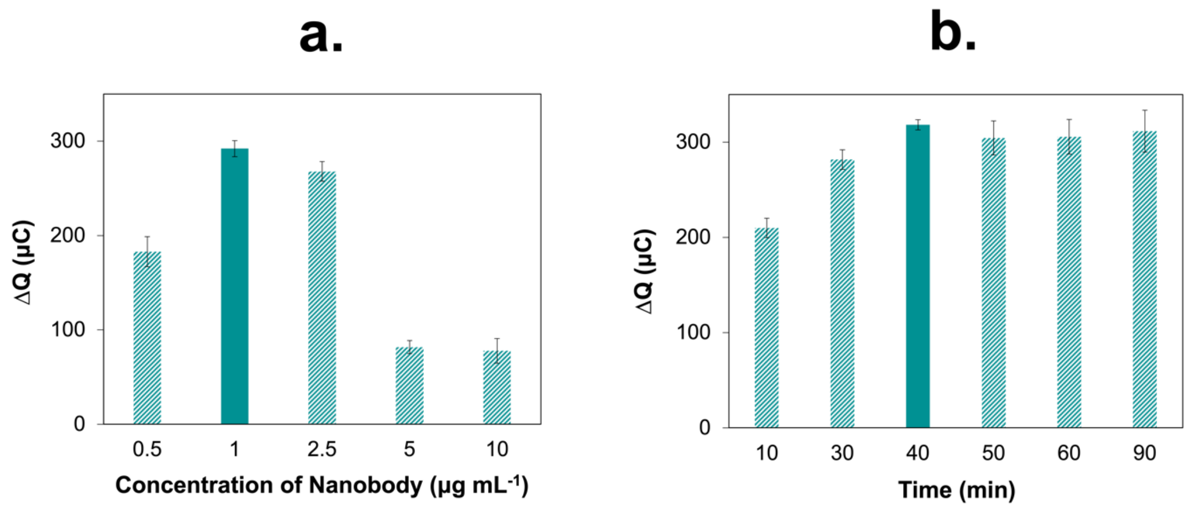

Figure S3. The optimization of assay conditions involved the following parameters: (a) concentration of nanobody and (b) binding time between nanobody and CRP target antigen.

2. Optimization of the immunoassay conditions

To achieve maximal sensing efficiency and optimal functionality of the microfluidic device, important parameters were thoroughly investigated. The nanobody served as the recognition element receptor for CRP, with a CRP concentration of 10 ng mL^{-1} being used. Additional parameters, such as the anodization potential and time, as well as the concentration of redox species (K_3FeCN_6), can be found in the previous work[1,4].

The optimal immunoassay conditions are as follows: 1.5 V vs Ag/AgCl of anodization potential, 120 s of anodization time, 25 mM of concentration of [$K_3Fe(CN)_6$], 1 μg mL^{-1} of nanobody, and 40 min of the binding time.

2.1 Concentration of nanobody

Further investigation was conducted to determine the optimal concentration of CRP nanobody specific to CRP. The nanobody concentration was varied within the range of 0.5 to 10 μg mL^{-1}. As shown in Fig. S3a, the maximum ΔQ was observed at nanobody concentration of 1 μg mL^{-1}. Beyond this range, the increased biomolecular density on the electrode surface could introduce steric hindrance, leading to an adverse impact on the electrochemical charge response. Hence, to reduce such effects, a concentration of 1 μg mL^{-1} of nanobody was therefore selected to be the most suitable for subsequent experiment.

2.2 Binding time

The effect of binding time between nanobody and CRP antigen, ranging from 10 to 90 min, was evaluated, as shown in Fig. S3b. Within the studied range, the maximal ΔQ value was obtained at 40 min. However, as the time was extended (> 40 min), a plateau signal of ΔQ was discovered, indicating a saturated state between nanobody as the recognition element and CRP antigen, which could form on the modified electrode. As a result, a binding time of 40 min was chosen for further studies.

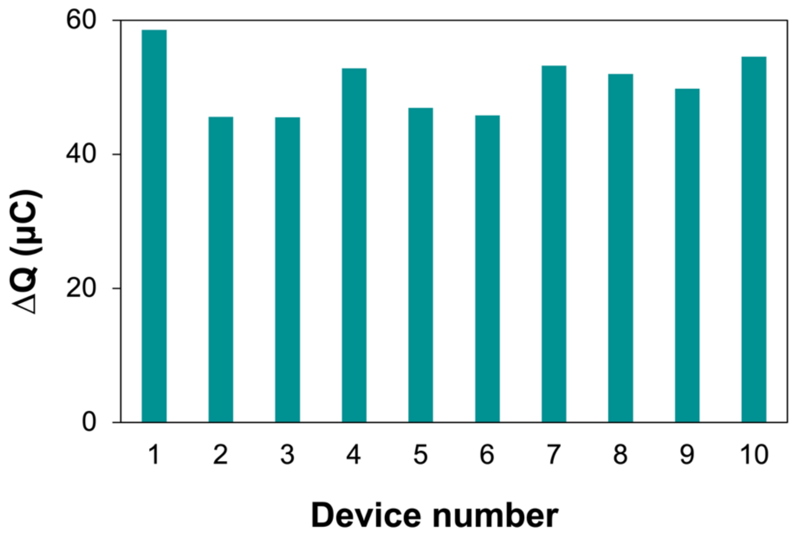

Figure S4 The reproducibility study of the proposed sensor.

135

Table S2 The analytical performance of the proposed microfluidic device for the detection of CRP in comparison with previous reports

Electrode	System	Electrochemical model	Receptor	Linear range (ng mL^{-1})	LOD (ng mL^{-1})	Stability (days)			Ref.
						RT	RT (close humid box)	Freezer	
Au electrode	Static	CHI660E	Peptide	0 – 36	0.7	Not specified	Not specified	12	[5]
ITO	Static	Autolab µAutolabIII	Peptide	1000 - 10000	340	Not specified	Not specified	Not specified	[6]
Au electrode	Static	Autolab	Peptide	0.5 nM – 10 nM	240 pM	Not specified	Not specified	Not specified	[7]
Au electrode	Static	Autolab PGSTAT204	Antibody	0.2 – 31.5	7	24	Not specified	Not specified	[8]
GCE	Static	CHI760E	Polymer	0.00001 - 1000	0.000041	Not specified	25	Not specified	[9]
GCE	Static	Autolab µAutolabIII	Bacteriophages	4000 – 100000	40	7	Not specified	Not specified	[10]
SPCE	Static	CHI660E	Polymer	1 - 1000000	0.3	Not specified	Not specified	5	[11]
SPE	Static	CHI660E	Aptamer	0.001 – 100	0.00002941	Not specified	Not specified	9	[12]
SPCE	Static	Autolab PGSTAT101	Polymer	5 – 5000	1.6	Not specified	Not specified	56	[13]
SPGE	Static	Autolab PGSTAT30	Antibody	50 – 100000	15	Not specified	Not specified	15	[14]
SPGE	Static	PalmSens	polymer	Not specified	500	7 hour	Not specified	Not specified	[15]
SPGE	Static	CHI660B	Antibody	1 – 10000	0.38	Not specified	Not specified	28	[16]
SPGE	Static	Autolab PGSTAT30	Antibody	5 – 500000	1	7	Not specified	Not specified	[17]
Microfluidic micromotors immunoassay	Flow	Metrohm µStat 800 multi potentiostat/ Galvanostat	Antibody	1000 – 100000	540	Not specified	Not specified	Not specified	[18]

Au electrode (*Analysis time 8 min)									
Lateral flow device SPE (Analysis time 17 min)	Flow	Autolab PGSTAT101	Antibody	1 – 10000	3	Not specified	Not specified	245	[19]
Capillary driven immunoassay SPGE (Analysis time 3 min)	Flow	PalmSens4	Antibody	1 – 1000	0.32	7	21	56	[4]
		PalmSens4	Peptide	0.1 – 1000	0.047	56	28	56	[4]
		PalmSens4	Peptide	10 – 100000	2.7	56	28	56	[4]
		PalmSens4	Nanobody	0.1 – 1000	0.0076	35	28	56	This work
		PalmSens4	Nanobody	10 – 100000	1.18				
		NFC	Nanobody	10 – 100000	1.79				

Abbreviations; Indium tin oxide (ITO), Glassy carbon electrode (GCE), Screen-printed electrode (SPE), Screen-printed carbon electrode (SPCE), Screen-printed graphene electrode (SPGE), electrochemical paper-based analytical devices (ePADs). *Noted that the analysis time was measured after the redox solution, or the buffer loaded in the flow system.

Table S3 Estimated cost breakdown of sensor flamework, electrode, and chemical and reagents for each sensor. *Noted that the production of the anti-CRP nanobodies is not included in the calculation.

	**Materials**	**Estimated cost/sample (Euro, €)**
**Sensor framework**	Transparent PET film	0.00477
	Hydrophilic film	0.00996
	Double-sided adhesive tape	0.00407
	Paper	0.05789
	**Subtotal**	0.06976
**Electrode**	Carbon-graphene ink	0.04500
	Ag/AgCl ink	0.00500
	**Subtotal**	**0.05000**
**Chemicals and reagents**	$NaIO_4$	0.00012
	LiCl	0.00060
	$NaCH_3CN$	0.00004
	Casein	0.01039
	PBS	0.00067
	$K_3[Fe(CN)_6]^{3-}$	0.00004
	**Subtotal**	**0.01186**
**Total cost**		0.13522

Table S4 The proposed microfluidic device with nanobody as the receptor was utilized for detecting CRP in artificially spiked serum.

Receptor	Spiked value (ng mL^{-1})	Detected Value (ng mL^{-1}) NFC	Recovery[a] (%)	Detected Value (ng mL^{-1}) PalmSens4	Recovery[b] (%)
Nanobody	0	0.013	-	0.011	-
	10	10.5 ± 1.9	104.8	10.8 ± 2.38	108.1
	75	68.6 ± 5.1	91.4	74.6 ± 6.24	99.4
	500	492.6 ± 0.4	98.5	482.8 ± 8.95	96.6

[a]Recovery calculated using NFC = (concentration of CRP found – concentration of CRP in the sample) / (concentration of CRP added) × 100%

[b]Recovery calculated using PalmSens4 = (concentration of CRP found – concentration of CRP in the sample) / (concentration of CRP added) × 100%

Table S5 Determination results by the proposed microfluidic device with traditional potentiostat (PalmSens4) for CRP detection in spiked whole blood samples.

No. of Sample	Spiked value (μg mL^{-1})	Detected Value (μg mL^{-1})	Recovery[a] (%)	Relative error[b] (%)
1	0	2.16	-	-
	0.5	2.62 ± 0.63	91.35	-8.65
	5	7.96 ± 1.34	115.9	+15.9
	25	26.58 ± 0.45	97.65	-2.35
2	0	6.4	-	-
	0.5	6.86 ± 2.60	93.78	-6.21
	5	11.62 ± 0.07	104.6	+4.58
	25	31.95 ± 0.57	102.2	+2.23
3	0	0.23	-	-
	0.5	0.78 ± 1.20	110.7	+10.7
	5	4.47 ± 3.11	84.74	-15.3
	25	26.3 ± 0.14	104.3	+4.33
4	0	1.11	-	-
	0.5	1.65 ± 5.04	109.9	+9.89
	5	7.07 ± 0.50	119.3	+19.35

	25	26.4 ± 0.22	101.1	+1.07
5	0	0.86	-	-
	0.5	1.42 ± 3.69	111.7	+11.7
	5	5.19 ± 3.33	86.52	-13.5
	25	24.0 ± 0.71	92.35	-7.64
6	0	2.87	-	-
	0.5	3.42 ± 2.26	110.6	+10.6
	5	8.47 ± 1.51	112.0	+12.0
	25	27.5 ± 0.04	98.6	-1.43
7	0	5.58	-	-
	0.5	6.0 ± 1.68	84.43	-15.6
	5	11.4 ± 1.99	116.4	+16.4
	25	25.6± 0.23	80.22	-19.8
8	0	1.51	-	-
	0.5	2.01 ± 2.00	100.2	+0.22
	5	5.55 ± 0.14	80.83	-19.2
	25	21.9 ± 3.90	81.63	-18.4
9	0	8.14	-	-
	0.5	8.61 ± 1.34	93.40	-6.60
	5	12.9 ± 1.30	94.89	-5.11
	25	36.7 ± 1.64	114.1	+14.1
10	0	8.22	-	-
	0.5	8.64 ± 0.11	83.54	-16.5
	5	13.70 ± 0.61	109.5	+9.46
	25	38.15± 0.76	119.7	+19.7

References:

(1) Boonkaew, S.; Yakoh, A.; Chuaypen, N.; Tangkijvanich, P.; Rengpipat, S.; Siangproh, W.; Chailapakul, O. An Automated Fast-Flow/Delayed Paper-Based Platform for the Simultaneous Electrochemical Detection of Hepatitis B Virus and Hepatitis C Virus Core Antigen. *Biosens. Bioelectron.* **2021**, *193,* 113543. https://doi.org/10.1016/j.bios.2021.113543.

(2) Beck, J. J.; Alenicheva, V.; Rahn, K. L.; Russo, M. J.; Baldo, T. A.; Henry, C. S. Evaluating the Performance of an Inexpensive, Commercially Available, NFC-Powered

and Smartphone Controlled Potentiostat for Electrochemical Sensing. *Electroanalysis* **2023**, *35* (6), e202200552. https://doi.org/10.1002/elan.202200552.

(3) Pungjunun, K.; Yakoh, A.; Chaiyo, S.; Siangproh, W.; Praphairaksit, N.; Chailapakul, O. Smartphone-Based Electrochemical Analysis Integrated with NFC System for the Voltammetric Detection of Heavy Metals Using a Screen-Printed Graphene Electrode. *Microchim. Acta* **2022**, *189* (5), 191. https://doi.org/10.1007/s00604-022-05281-x.

(4) Boonkaew, S.; Szot-Karpińska, K.; Niedziółka-Jönsson, J.; Pałys, B.; Jönsson-Niedziółka, M. Point-of-Care Testing for C-Reactive Protein in a Sequential Microfluidic Device. *Sens. Actuators B Chem.* **2023**, *397,* 134659. https://doi.org/10.1016/j.snb.2023.134659.

(5) Yang, H. J.; Kim, M. W.; Raju, C. V.; Cho, C. H.; Park, T. J.; Park, J. P. Highly Sensitive and Label-Free Electrochemical Detection of C-Reactive Protein on a Peptide Receptor−gold Nanoparticle−black Phosphorous Nanocomposite Modified Electrode. *Biosens. Bioelectron.* **2023**, *234*, 115382. https://doi.org/10.1016/j.bios.2023.115382.

(6) Szot-Karpińska, K.; Kudła, P.; Orzeł, U.; Narajczyk, M.; Jönsson-Niedziółka, M.; Pałys, B.; Filipek, S.; Ebner, A.; Niedziółka-Jönsson, J. Investigation of Peptides for Molecular Recognition of C-Reactive Protein–Theoretical and Experimental Studies. *Anal. Chem.* **2023**. https://doi.org/10.1021/acs.analchem.3c03127.

(7) Piccoli, J. P.; Soares, A. C.; Oliveira, O. N.; Cilli, E. M. Nanostructured Functional Peptide Films and Their Application in C-Reactive Protein Immunosensors. *Bioelectrochemistry* **2021**, *138,* 107692. https://doi.org/10.1016/j.bioelechem.2020.107692.

(8) Kanyong, P.; Catli, C.; Davis, J. J. Ultrasensitive Impedimetric Immunosensor for the Detection of C-Reactive Protein in Blood at Surface-Initiated-Reversible Addition–Fragmentation Chain Transfer Generated Poly(2-Hydroxyethyl Methacrylate) Brushes. *Anal. Chem.* **2020**, *92* (7), 4707–4710. https://doi.org/10.1021/acs.analchem.9b05030.

(9) Cui, M.; Che, Z.; Gong, Y.; Li, T.; Hu, W.; Wang, S. A Graphdiyne-Based Protein Molecularly Imprinted Biosensor for Highly Sensitive Human C-Reactive Protein Detection in Human Serum. *Chem. Eng. J.* **2022**, *431,* 133455. https://doi.org/10.1016/j.cej.2021.133455.

(10) Szot-Karpińska, K.; Kudła, P.; Szarota, A.; Narajczyk, M.; Marken, F.; Niedziółka-Jönsson, J. CRP-Binding Bacteriophage as a New Element of Layer-by-Layer Assembly Carbon Nanofiber Modified Electrodes. *Bioelectrochemistry* **2020**, *136,* 107629. https://doi.org/10.1016/j.bioelechem.2020.107629.

(11) Cheng, Y.-Y.; Feng, X.-Z.; Zhan, T.; An, Q.-Q.; Han, G.-C.; Chen, Z.; Kraatz, H.-B. A Facile Indole Probe for Ultrasensitive Immunosensor Fabrication toward C-Reactive Protein Sensing. *Talanta* **2023**, *262*, 124696. https://doi.org/10.1016/j.talanta.2023.124696.

(12) Gao, H.; Bai, Y.; He, B.; Tan, C. S. A Simple Label-Free Aptamer-Based Electrochemical Biosensor for the Sensitive Detection of C-Reactive Proteins. *Biosensors* **2022**, *12* (12). https://doi.org/10.3390/bios12121180.

(13) Pinyorospathum, C.; Chaiyo, S.; Sae-ung, P.; Hoven, V. P.; Damsongsang, P.; Siangproh, W.; Chailapakul, O. Disposable Paper-Based Electrochemical Sensor Using Thiol-Terminated Poly(2-Methacryloyloxyethyl Phosphorylcholine) for the Label-Free Detection of C-Reactive Protein. *Microchim. Acta* **2019**, *186* (7), 472. https://doi.org/10.1007/s00604-019-3559-6.

(14) Boonkaew, S.; Chaiyo, S.; Jampasa, S.; Rengpipat, S.; Siangproh, W.; Chailapakul, O. An Origami Paper-Based Electrochemical Immunoassay for the C-Reactive Protein Using a Screen-Printed Carbon Electrode Modified with Graphene and Gold Nanoparticles. *Microchim. Acta* **2019**, *186* (3), 153. https://doi.org/10.1007/s00604-019-3245-8.

(15) Baradoke, A.; Hein, R.; Li, X.; Davis, J. J. Reagentless Redox Capacitive Assaying of C-Reactive Protein at a Polyaniline Interface. *Anal. Chem.* **2020**, *92* (5), 3508–3511. https://doi.org/10.1021/acs.analchem.9b05633.

(16) Boonkaew, S.; Jang, I.; Noviana, E.; Siangproh, W.; Chailapakul, O.; Henry, C. S. Electrochemical Paper-Based Analytical Device for Multiplexed, Point-of-Care Detection of Cardiovascular Disease Biomarkers. *Sens. Actuators B Chem.* **2021**, *330*, 129336. https://doi.org/10.1016/j.snb.2020.129336.

(17) Boonyasit, Y.; Chailapakul, O.; Laiwattanapaisal, W. A Folding Affinity Paper-Based Electrochemical Impedance Device for Cardiovascular Risk Assessment. *Biosens. Bioelectron.* **2019**, *130*, 389–396. https://doi.org/10.1016/j.bios.2018.09.031.

(18) Molinero-Fernández, Á.; López, M. Á.; Escarpa, A. Electrochemical Microfluidic Micromotors-Based Immunoassay for C-Reactive Protein Determination in Preterm Neonatal Samples with Sepsis Suspicion. *Anal. Chem.* **2020**, *92* (7), 5048–5054. https://doi.org/10.1021/acs.analchem.9b05384.

(19) Petruzzi, L.; Maier, T.; Ertl, P.; Hainberger, R. Quantitative Detection of C-Reactive Protein in Human Saliva Using an Electrochemical Lateral Flow Device. *Biosens. Bioelectron. X* **2022**, *10*, 100136. https://doi.org/10.1016/j.biosx.2022.100136.

95
